# Tissue Engineering for Rotator Cuff Repair: An Evidence-Based Systematic Review

**DOI:** 10.1155/2012/418086

**Published:** 2011-11-10

**Authors:** Nicola Maffulli, Umile Giuseppe Longo, Mattia Loppini, Alessandra Berton, Filippo Spiezia, Vincenzo Denaro

**Affiliations:** ^1^Centre for Sports and Exercise Medicine, Barts and the London School of Medicine and Dentistry, Mile End Hospital, 275 Bancroft Road, London E1 4DG, UK; ^2^Department of Orthopaedic and Trauma Surgery, Campus Bio-Medico University, Via Alvaro del Portillo 200, Trigoria, 00128 Rome, Italy; ^3^Centro Integrato di Ricerca (CIR), Campus Bio-Medico University, Via Alvaro del Portillo 21, 00128 Rome, Italy

## Abstract

The purpose of this systematic review was to address the treatment of rotator cuff tears by applying tissue engineering approaches to improve tendon healing, specifically platelet rich plasma (PRP) augmentation, stem cells, and scaffolds. Our systematic search was performed using the combination of the following terms: “rotator cuff”, “shoulder”, “PRP”, “platelet rich plasma”, “stemcells”, “scaffold”, “growth factors”, and “tissue engineering”. No level I or II studies were found on the use of scaffolds and stem cells for rotator cuff repair. Three studies compared rotator cuff repair with or without PRP augmentation. All authors performed arthroscopic rotator cuff repair with different techniques of suture anchor fixation and different PRP augmentation. The three studies found no difference in clinical rating scales and functional outcomes between PRP and control groups. Only one study showed clinical statistically significant difference between the two groups at the 3-month follow up. Any statistically significant difference in the rates of tendon rerupture between the control group and the PRP group was found using the magnetic resonance imaging. The current literature on tissue engineering application for rotator cuff repair is scanty. Comparative studies included in this review suggest that PRP augmented repair of a rotator cuff does not yield improved functional and clinical outcome compared with non-augmented repair at a medium and long-term followup.

## 1. Introduction

Rotator cuff tears are an important cause of shoulder pain and disability [1]. Despite its frequency and great health care costs in industrialised countries, the best management options for rotator cuff tears are still debated [1, 2]. One of the reasons is that the pathogenesis of rotator cuff tears is still largely unknown [3–11]. Moreover, the cuff has a limited ability to heal back to its insertion on the humerus after the repair process is ended. Given this limited ability for healing [12], novel biomechanical strategies (double-row techniques [13–15]) and biological augmentations (such as growth factors and cytokines, platelet rich plasma (PRP) [16], gene therapy [17], tendon graft [18, 19], and tissue engineering with mesenchymal stem cells [17]) have been proposed to enhance rotator cuff tendon healing. They hold the promise to yield more successful outcomes for the management of patients with tendon pathology [1, 2, 11, 20–52]. 

The purpose of this systematic review was to address the treatment of rotator cuff tears by applying tissue engineering approaches to improve tendon healing.

## 2. Methods

We identified all published studies in the English language addressing tissue engineering for rotator cuff repair, using a methodology already validated in our setting [1, 2, 5, 11, 12, 15, 17–19, 23, 24, 27, 29, 32–34, 38–40, 42–46, 53–75]. Two independent reviewers performed a search of the Medline database on PubMed, CINAHL (Cumulative Index to Nursing and Allied Health Literature), EMBASE, and the Cochrane Central Register of Controlled Trials from inception of database to July 2011, using the combination of following terms: “rotator cuff”, “shoulder”, “PRP”, “platelet rich plasma”, “stem cells”, “scaffold”, “growth factors”, and “tissue engineering”. Before conducting the literature search, we established the study design and specific objectives. Studies were included in our systematic review if they met the following guidelines: (1) they provided level I-II evidence addressing the area of interest outlined above, (2) they included measures of functional and clinical outcome, (3) they had minimum 3 month followup, and (4) they were published in peer review journal. Citations from relevant studies, as well as from any review articles captured by the search, were also examined to determine if they were suitable for inclusion. Studies not meeting these guidelines were excluded. Patient demographic information, rotator cuff tear features, surgical techniques, objective and subjective outcome measurements, radiological examinations, and complications were extracted from the studies. The objectives were to evaluate the clinical and structural outcomes of patients receiving tissue engineering strategies compared to control group patients.

## 3. Data Abstraction

The data were independently extracted by three reviewers from each of the selected studies. The demographic data collected included the type of study, level of evidence, number of patients enrolled, age, gender, and mean followup. The collected features of rotator cuff tears included tear size according to the classification of DeOrio and Cofield [76] (small: <1 cm; medium: 1 to 3 cm; large: 3 to 5 cm; massive: >5 cm) or arthroscopic classification of tear retraction (grade 1: the tear edge is lying over the greater tuberosity; grade 2: the tear exposed the humeral head without retraction to the glenoid; grade 3: the tear is extended to the glenoid; grade 4: the tear is retracted medial to the glenoid). 

Surgical technique data were also recorded, including the surgical repair procedure, number and type of anchors, type of arthroscopic knot, suture type, and concomitant procedures.

Preoperative and postoperative data included range of motion; strength, evaluated in terms of strength in external rotation (SER) and clinical outcome scales (Constant [77]; University of California, Los Angeles-UCLA [78]; American Shoulder and Elbow Surgeons-ASES [79]; Disabilities of the Arm, Shoulder and Hand-DASH; Shoulder Pain and Disability Index-SPADI; Simple Shoulder Test-SST; Visual Analog Score for Pain-VAS).

Postoperative imaging modality and outcome (complete healing, partial healing, and no healing) were also analyzed. The complications related to the surgical procedures and the biological augmentations were also recorded.

## 4. Results

**Figure 1:**
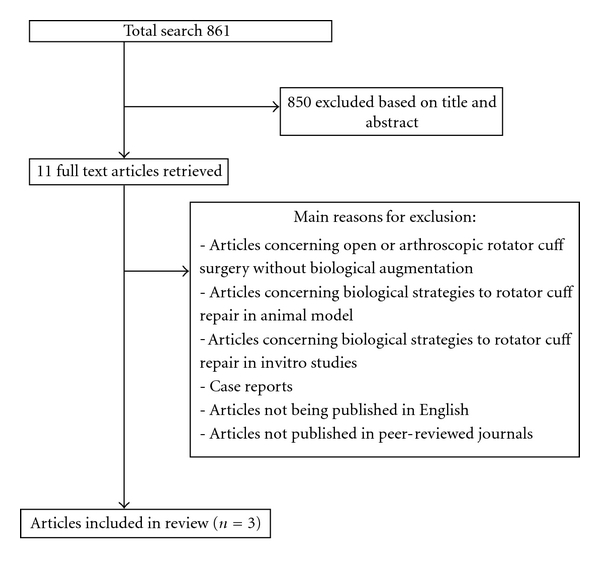
Flowchart of the search strategy and selection of articles.

The search strategy identified 861 articles. Evaluation of title and abstract left 11 articles to be evaluated. Full text of all the eligible papers was screened for inclusion and exclusion criteria, leading to 3 studies on PRP augmentation included in the review [80–82]. No clinical studies on application of stem cells and scaffolds for rotator cuff repair were found. The study selection process and reasons for exclusions are summarized in Figure 1. Of the three included studies, one level I study evaluated patients with rotator cuff tear in whom the repair was augmented with membrane of platelet-rich fibrin matrix [80], one level I study with PRP and autologous thrombin [82] and one level II study with PRP gel [81].

## 5. Patient Demographics

There were 2 randomized controlled trials (Level I) [80, 82] and 1 prospective cohort study (Level II) [81] (Table 1). In 2 studies, the followup was completed by 100% of patients [80, 81], whereas in 1 study it was completed by 85% of patients [82]. The mean age of patients ranged between 55 and 60 years in both PRP and control group for all the studies. Each study compared the study groups. No statistically significant differences were found in terms of age, gender, and followup [80–82].

## 6. Surgical Technique

All the studies described the surgical procedure consisting of arthroscopic rotator cuff repair with suture anchor fixation (Table 2). In all the studies, the number of suture anchors was established according to the size of rotator cuff lesion in both control and PRP group. Suture anchors ranged from 1 to 3 in patients with small or medium tears and from 3 to 5 for large or massive tears. None of the studies performed statistical analysis comparing the mean number of anchors between the two groups. In two studies, the authors used bioabsorbable suture anchors [81, 82] and metallic suture anchors in the other study [80]. Rotator cuff repair was performed with different arthroscopic techniques. Castricini et al. [80] performed a double-row technique with metal suture anchors (Fastin RC Anchor; DePuy Mitek, Raynham, Massachusetts) in which medial row was secured using nonsliding knots in a mattress configuration, whereas lateral row used sliding knots with 3 alternating half hitches. Randelli et al. [82] performed a single-row technique with absorbable suture anchors (Bio-Corkscrew; Arthrex, Naples, FL, USA). Jo et al. [81] performed a suture bridge technique with absorbable suture anchors (Bio-Corkscrew; Arthrex, Naples, FL, USA) in which medial row was secured using a slippage proof knot, whereas lateral row was secured using PushLocks (Arthrex) or suture anchors.

In addition to rotator cuff repair, concomitant procedures were performed in both groups in all the studies. In the study by Castricini et al. [80], 25 patients in the control group (56%) underwent acromionplasty, 22 (49%) underwent a biceps tenodesis, and 5 underwent a biceps tenotomy (11%); 12 patients (28%) underwent at acromionplasty, 21 (49%) underwent a biceps tenodesis, and 3 (7%) underwent a biceps tenotomy in the PRP group. Randelli et al. [82] performed an acromionplasty in 27 patients (100%), biceps tenodesis in one patient (4%), and biceps tenotomy in 18 patients (67%) in the control group. They performed acromionplasty in 26 patients (100%), biceps tenodesis in 4 patients (15%), and biceps tenotomy in 15 patients (58%) in the PRP group. Jo et al. [81] rarely performed an acromionplasty: 4 patients (17%) in the control group and 3 patients (16%) in the PRP group.

The PRP augmentation of the rotator cuff was performed with different techniques. Castricini et al. [80] used a platelet-rich fibrin matrix (PRFM) which was a flat membrane of antilogous suturable fibrin. It was applied under the supraspinatus tendon, above the bleeding surface of the greater tuberosity, by using one of the suture limbs of lateral anchors and by pulling the other end of the suture. Randelli et al. [82] used activated PRP combined with antilogous thrombin, which was loaded with syringes. They injected this product between the bone and the repaired rotator cuff and then performed a dry arthroscopic check of the clot formation. Jo et al. [81] used PRP gel. In each patient, three PRP gels were placed in the repair site at the tendon-bone interface during the arthroscopic repair procedure. When the PRP gels were in place, medial and lateral row sutures were tied, and PRP gels were snuggled between the repaired tendon and the bone insertion.

## 7. Rehabilitation Protocol

The postoperative rehabilitation was the same for the control group and the PRP group in each study, limiting performance bias. A rest period was performed in all the studies. Castricini et al. [80] performed 3 weeks of immobilization using a sling with an abduction pillow. Jo et al. [81] performed 4 weeks of immobilization for small to large tears, and 6 weeks for massive tears, using an abduction brace. Randelli et al. [82] performed a short rest period of 10 days wearing the sling.

During the rest period, Castricini et al. [80] allowed only pendulum exercises, whereas Jo et al. [81] allowed shrugging, protraction, and retraction of shoulder girdles: mobilization of the elbow, wrist, and hand; and external rotation of the arm to neutral according to patient compliance. Passive range of motion (ROM) and active-assisted ROM exercises were allowed after 3 to 6 week rest period, according to author protocols [80, 81]. 

In the study by Randelli et al. [82], patients started passive assisted exercises after the rest period to obtain a complete passive ROM restoration. At 30 days from surgery, assisted active range-of-motion exercises were allowed. 

Strengthening exercises of the rotator cuff and scapular stabilizers were performed after 6–8 weeks [80, 82] or 12 weeks [81], according to author protocols. Light sports activities were allowed 3 months after surgery, whereas full return to sports, overhead activities, and heavy manual work were allowed after a minimum of 6 months, based on patient recovery [80, 81].

## 8. Clinical Shoulder Scores

All the studies used the Constant score, and 2 used the UCLA and SST scores [81, 82]. In addition, Randelli et al. [82] used also SER and VAS scores, whereas Jo et al. [81] used ASES, DASH, and SPADI scores (Table 3). 

Castricini et al. [80] found a statistically significant improvement from the preoperative to postoperative mean values in the Constant score for each group (*P* = 0.001), but no statistically significant differences when comparing the 2 groups.

In the study by Randelli et al. [82], in both groups, postoperative values of Constant, UCLA, and SST scores significantly improved in comparison to the preoperative values at 3 months after surgery. There was a statistically significant difference between the PRP and control groups for all clinical outcomes at the 3-month followup (Constant, *P* = 0.02; UCLA, *P* = 0.03; SST, *P* = 0.02). However, no significant differences between two groups were found at 6, 12, and 24 month followup.

In the study by Jo et al. [81], preoperative values were similar between two groups for all functional scores. Postoperative values of all scores showed a progressive improvement in both groups. ASES, Constant, and SPADI scores were significantly higher in the control group compared with the PRP group at 3 months after surgery (Table 3). However, no significant differences between two groups were observed for any of these scoring systems at 6, 12, and 24 months of followup.

## 9. Strength and Range of Motion

Only one study provided strength measurements [82]. Authors measured the strength in external rotation (SER) in a sitting position with the arm at side (neutral position).

In the control group, SER score values started to increase at 6 months after surgery. Only at the last followup, there was a statistically significant difference between preoperative (2.3 kg ± 2 kg) and postoperative values (4 kg ± 1.9 kg) (*P* = 0.01). On the other hand, a statistically significant improvement of SER score was found at the first followup in the PRP group (from 1.9 kg ± 1.7 kg to 3 kg ± 1.6 kg; *P* = 0.003). The SER postoperative values increased until 6 months after surgery (*P* < 0.001), while at the last followup any significant improvement was recorded. 

However, there were no differences in strength measurements when comparing the results of control and PRP groups at 6, 12, and 24 months of followup.

Only in one study the evaluation of range of motion (ROM) was performed [81]. Before surgery, any difference of ROM between two groups was found. ROM decreased in the early postoperative period. Then, starting from 3 months after surgery, ROM increased gradually until final followup. At final followup, forward flexion, and abduction improved significantly in both groups (*P* = 0.001); internal rotation improved significantly only in the PRP group (*P* = 0.033); external rotation did not improve in either group (*P* > 0.05). No statistically significant difference in ROM was found at 3-, 6-, or 12-month followup.

## 10. Pain

Two studies performed an assessment of pain, expressed in terms of VAS score [81, 82]. In the study by Randelli et al. [82], the baseline values of VAS were significantly different between two groups. In the control group, postoperative values were significantly lower compared with preoperative values, starting from day 7 after surgery (*P* = 0.003). On the other hand, PRP group showed a statistically significant reduction of mean VAS score as soon as day 3 after surgery (*P* = 0.04).

The VAS score was significantly lower in the PRP group at 3, 7, 14, and 30 days of followup (Table 3). Moreover, a significant difference was found between the two groups at 24-month followup (*P* = 0.002).

In the study by Jo et al. [81], preoperative VAS scores were similar in the two groups. The reduction of postoperative values was significant and gradual over time until final followup in both groups (all *P* = 0.001). However, there were no significant differences between the two groups for any value at any time point of followup (all *P* > 0.05).

## 11. Radiological Assessment

All the studies included postoperative magnetic resonance imaging (MRI) to evaluate tendon integrity. Castricini et al. [80] performed MRI at a mean of 20.2 months from surgery for both the control group and the PRP group. Although, the authors reported a higher rate of tendon rerupture in the control group compared with the PRP group (10.5% versus 2.5%), the difference between arthroscopic repair with or without PRFM was not statistically significant (*P* = 0.07). Randelli et al. [82] performed MRI at a mean of 23 ± 5 months from surgery (25 ± 5 months for the control group and 21 ± 5 months for the PRP group). The mean radiological followup time was slightly longer in the control group (*P* = 0.003). Authors found a not statistically significant difference between the rates of tendon rerupture in the control group compared with the PRP group (52% versus 40%, resp.; *P* = 0.4). In the study by Jo et al. [81], the mean time between surgery and postoperative MRI was 13.93 ± 4.23 in the PRP group and 15.29 ± 5.6 in the conventional group (*P* = 0.449). Authors reported a higher overall retear rate in the control group (41.2%) than in the PRP group (26.7%), without any statistically significant difference (*P* = 0.388).

## 12. Complications

No complications related to the use of PRP were reported in the included studies.

## 13. Discussion

The current literature on tissue engineering application for rotator cuff repair is scanty. Although several authors advocate it, uncertainty still exists as to whether tissue engineering is able to yield improved results. Our review suggests that patients receiving PRP augmentation for rotator cuff repair do not show improved functional outcomes when compared with a nonaugmented repair at medium and long-term followup. At a short-term followup, patients managed with PRP augmented repair showed better control of post-operative pain [82]. On the other hand, the structural integrity of the rotator cuff seemed to be slightly better in the PRP augmented group, even though the small number of patients in the included studies did not allow definitive conclusions. Even though no results on the costs of PRP surgery were available from the included studies, it is possible to speculate that PRP augmented rotator cuff repair yielded to increased economic costs, both for the duration of surgery and the cost for PRP preparation. However, these aspects need to be evaluated in future studies.

## 14. Selection Bias

Two of the studies included in this systematic review were randomized controlled trial [80, 82] and one was a cohort study [81] (Levels I to II). The random allocation of patients into two groups, receiving PRP treatment or not, should dramatically limit bias. In the study by Jo et al. [81], patients were informed about the use of PRP before surgery and decided themselves whether to have PRP placed at the time of surgery. Generally the 2 groups showed similar age, sex, dominance, symptom duration, and aggravation period before surgery, thus limiting the potential for selection bias.

The factors that have been shown to affect clinical outcome including age, gender, rotator cuff tear size, and acromioclavicular joint pathology were similar between groups in all the studies. In the study by Randelli et al. [82], 11 patients in the PRP group and 13 patients in the control group had only lesions of the supraspinatus, 6 patients in the PRP group and 4 patients in the control group had all three tendons involved. In the study by Jo et al. [81] there were no significant differences in anteroposterior and mediolateral tear sizes between the 2 groups, and rotator cuff muscle status evaluated using global fatty degeneration indices [83], modified tangent signs, and occupational ratios [84] were also not significantly different. Several studies in the open, mini-open, and arthroscopic literature showed that tear size is an important determinant of outcome and healing [85–90]. 

Three studies reported no difference in clinical rating scales between groups. In the study by Jo et al. [81] the addition of PRP gel to arthroscopic rotator cuff repair was not found to accelerate the relief of pain; the recovery of ROM, strength, or function; or improve overall satisfaction as compared with conventional repair at any time point. Rather, the recovery of some measures in the PRP group, such as ASES, Constant, and SPADI functional scores, and abduction were slower than in the conventional group at 3 months after surgery [81]. The only significant improvement found in the PRP group was in internal rotation at final followup [81].

Randelli et al. [82] found statistically significant difference between the PRP and control groups for all the clinical outcomes (Constant, SER, UCLA, SST) at 3-month followup, but no significant differences between the PRP and control groups at 6, 12, and 24 months. Moreover, the pain score in the treatment group was lower than the control group at 3, 7, 14, and 30 days after surgery, but there was no difference between the 2 groups after 6, 12, and 24 months. 

No studies showed significant difference in postoperative tendon healing. Castricini et al. [80] found no difference in tendon thickness and footprint size between the 2 groups. The only difference between the 2 groups was in tendon signal, whose significance was of difficult interpretation. Randelli et al. [82] found no significant difference in the MRI healing rate of the rotator cuff. The number of identified retears was 9 (40%) in the PRP group and 12 (52%) in the control group. This difference was not statistically significant. Retear rate was influenced by age, tear severity, and grade of retraction in the PRP group. Jo et al. [81] also found no significant improvement in structural integrity, and no significant difference in retear rates between the groups.

## 15. Performance Bias

Surgical technique was adequately described in all the studies [80–82]. Castricini et al. [80] used a double-row technique, Randelli et al. [82] used a single-row technique and Jo et al. [81] used a suture bridge technique. Performance bias may occur in studies where a disproportionate number of concomitant procedures are performed, but bias is largely limited because of homogeneity between groups. Rehabilitation protocol is another potential variable that may influence performance bias, but the same rehabilitation was implemented for each group in a single study. It was described in details in all the 3 studies [80–82].

## 16. Exclusion Bias

Castricini et al. [80] reported at last 16-month clinical results for all the patients (88) and radiological results for 78. In the study by Randelli et al. [82], of the 53 randomized participants, 45 completed clinical and radiological followup. Eight patients (4 for the treatment group and 4 for the control group) did not return at the final followup, and one patient in the PRP group died at about 1 year after the surgical intervention from cardiac arrest.

## 17. Detection Bias

All studies assessed clinical outcomes according to functional scores. The functional scoring systems used were Constant score, UCLA, ASES, SST, DASH, SPADI. All of these outcome scores have been validated as shoulder-specific outcome instruments [77–79]. All of the studies reported significant improvement between baseline and postoperative scores for each group. 

Three studies detect no significant difference in clinical rating scales between the PRP group and the control group. However, Randelli et al. [82] detected a significant improvement in the Constant, SER, UCLA, SST between the PRP and control groups at the 3-month followup. The VAS score was found to be significantly lower in the PRP group at 3, 7, 14, and 30 days postoperative.

All the studies used postoperative MRI. Each study performed statistical analysis between the PRP group and control group. 

Castricini et al. [80] reported the findings as tendon thickness, size of tendon footprint and intensity of the signal, grading each of these parameters on a scale from I to III. Randelli et al. [82] differentiated only between retear and intact tendon. Jo et al. [81] used Sugaya's method [91] for evaluation of structural integrity: Types I, II, and III were considered healed, types IV and V were considered retears. 

None of the studies reported a statistically significant improvement in the structural appearance with the PRP augmentation repair compared with the arthroscopic rotator cuff repair without augmentation. In the study by Randelli et al. [82], the number of identified retears was 9 (40%) in the PRP group and 12 (52%) in the control group, but this difference was not statistically significant. The repair integrity of the overall sample was significantly associated with age, shape, and tear retraction. The effect of prognostic factors was more evident in the PRP group. Also, in the study by Jo et al. [81] the overall retear rates between the 2 groups was not significantly different (8 cases (26.7%) in the PRP group and 14 (41.2%) in the conventional group).

These findings raise the debated question of PRP ability to improve tendon healing after rotator cuff repair. Experimental evidences indicates that PRP and growth factors aid tendon healing [92, 93]. This is the main concept behind the placement of PRP between bone and the torn end of a rotator cuff. However clinical studies failed to demonstrate significant improvement in the structural integrities of repaired tendons. Only the study by Randelli et al. [82] described accelerated healing in term of higher subjective scores (including daily living activities) at 3 months postoperative in the PRP group. Longer followup did not result in significant improvement of shoulder function or structural outcome. Reasons for this statistical insignificance were sought in nonoptimal concentration, activation status, or dose of PRP grow factors. Given the heterogeneity of PRP preparation products available on the market, it is possible that some preparations may be more effective than others. Future studies should be adequate in terms of standardization and characterization of the preparation of PRP to allow comparison of results. Tear severity has been advocated as another possible factor influencing studies results. However, preliminary results on this aspect are discordant. Randelli et al. [82] reported significant differences in some outcome measures at long-term followups in patients with stage 1 or 2 cuff tears. 

A limitation of our review is the small number of available studies on the topic. Interest in PRP is increasing but researches are still ongoing. Only 3 studies have been recently published on PRP use for rotator cuff repair. Sample sizes are relatively small (53 [82], 88 [80], 42 [81] patients, resp.). PRP device was different between the included studies. However, it was always positioned at the bone to tendon interface.

## 18. Conclusions

In conclusion, the current literature on tissue engineering application for rotator cuff repair is scanty [19, 94–97]. Comparative studies included in this review suggest that PRP augmented repair of a rotator cuff does not yield improved functional and clinical outcome compared with nonaugmented repair at medium and long-term followup. At a short-term followup, patients managed with PRP augmented repair showed better control of postoperative pain [3, 4, 16, 57, 58, 98–103]. The structural integrity of the rotator cuff seemed to be slightly better in the PRP augmented group, even though the small number of patients in the included studies did not allow definitive conclusions [8, 9, 12, 61–63, 104–109]. Relatively few studies, as well as small sample size, were the primary limitations of this systematic review [13, 14, 51, 52, 110–133]. Randomized, prospective trials are needed for more definitive answers.

## Figures and Tables

**Table 1:** Study and demographic data.

Study	Level of evidence		Patients	Mean age (Range or ± SD), y	Men*∖*Women	Followup (Range or ± SD), months	Size of lesion			
Castricini et al. [80], 2011	I	Control group	45	55.2 (37–69)	23/22	20.2 (16–30)	20 Small RCT	25 Medium RCT	0	0
		PRP group	43	55.5 (41–72)	17/26		18 Small RCT	25 Medium RCT	0	0

Randelli et al. [82], 2011	I	Control group	27	59.5 (±10.7)	13*∖*14	23	12 Minor	7 Moderate	4 Severe	4 Massive
		PRP group	26	61.6 (±8.3)	8*∖*18		9 Minor	7 Moderate	3 Severe	7 Massive

Jo et al. [81], 2011	II	Control group	23	59.80 (±8.84)	9*∖*14	20.30 (±1.89)	2 Small RCT	15 Medium RCT	3 Large RCT	3 Massive RCT
		PRP group	19	61.80 (±8.86)	6*∖*13	18.94 (±1.63)	1 Small RCT	7 Medium RCT	5 Large RCT	6 Massive RCT

RCT: rotator cuff tear.

**Table 2:** Surgical techniques and concomitant procedures.

Study		Surgical technique	Total *N* of anchors	Type of anchors	Type and size of suture	Type of knots	Concomitant procedures	Complications
Castricini et al. [80], 2011	Control group	Arthroscopic RC repair with double-row technique	2 for each patients	Metal suture anchors (Fastin RC Anchor; DePuy Mitek)	No. 2 Ethibond Excel (Ethicon)	Medial sutures: nonsliding knot in a mattress configuration; lateral sutures: sliding knot	Acromionplasty 25; Tenodesis 22; Tenotomy 5	0
	PRP group	Arthroscopic RC repair with double-row technique with membrane of PRFM augmentation	2 for 41 patients and 3 for 2 patients				Acromionplasty 12; Tenodesis 21; Tenotomy 3	0

Randelli et al. [82], 2011	Control group	Arthroscopic RC repair with single-row technique	1.6 ± 0.7	Absorbable suture anchors (Bio-Corkscrew; Arthrex)	—	—	Acromionplasty 27; Tenodesis 1; Tenotomy 18	0
	PRP group	Arthroscopic RC repair with single-row technique and injection of PRP and autologous thrombin	2 ± 0.9		—	—	Acromionplasty 26; Tenodesis 4; Tenotomy 15	0

Jo et al. [81], 2011	Control group	Arthroscopic RC repair with suture bridge technique	2 or 3 for small/medium tears; 3 to 5 for large/massive tear	Absorbable suture anchors (Bio-Corkscrew; Arthrex); PushLocks (Arthrex)	No. 1 Polydioxanone II suture (Ethicon)	Medial sutures: slippage proof knot; knotless suture anchor repair	Acromionplasty 4	0
	PRP group	Arthroscopic RC repair with suture bridge technique and application of PRP gel					Acromionplasty 3	0

RC: rotator cuff; PRFM: platelet-rich fibrin matrix.

**Table 3:** Clinical outcomes.

Study	Outcome measures		Pre-op.	3 days	1 month	3 months	6 months	12 months	16 months	24 months	
	Constant score										*P* value
Castricini et al., 2011 [80]		Control group	42.9 (22–55)						88.4 (54–100)		<0.001
		PRP group	42 (30–53)						88.4 (72–99)		<0.001
		*P* value							0.44		

Randelli et al., 2011 [82]	Constant score										
		Control group	42.2 ± 15.2			57.8 ± 11	72.3 ± 12.6	75.7 ± 9.5		78.7 ± 10	
		PRP group	44 ± 16.5			65 ± 9	73.1 ± 8.7	78.3 ± 6.4		82.4 ± 6.3	
		*P* value	0.6			0.02	0.7	0.3		0.1	
	UCLA										
		Control group	14.5 ± 5.6			24.2 ± 4.9	29.2 ± 4.9	31 ± 4.1		31.3 ± 4.1	
		PRP group	15.3 ± 5.9			26.9 ± 3	30.6 ± 4.1	31.2 ± 5.2		33.3 ± 2.2	
		*P* value	0.6			0.03	0.3	0.7		0.06	
	SER (Kg)										
		Control group	2.3 ± 2			2.1 ± 1.3	3.3 ± 1.3	3.7 ± 1.5		4 ± 1.9	
		PRP group	1.9 ± 1.7			3 ± 1.6	3.9 ± 2.1	4.2 ± 2.8		4.3 ± 2.3	
		*P* value	0.4			0.04	0.2	0.5		0.5	
	SST										
		Control group	4.7 ± 2.8			7.1 ± 2.7	10.5 ± 2.3	10.6 ± 1.5		10.9 ± 1.4	
		PRP group	4.8 ± 3.1			8.9 ± 2.2	10.6 ± 1.4	11.1 ± 0.9		11.3 ± 0.9	
		*P* value	0.9			0.02	0.9	0.3		0.3	
	VAS										
		Control group	6.4 ± 2	6.3 ± 2.8	2.4 ± 2.6						
		PRP group	4.8 ± 2	4 ± 3.2	1.1 ± 2.2						
		*P* value	0.003	0.007	0.01						

Jo et al. [81], 2011	Constant Score										
		Control group	50.78 ± 16.04			46.10 ± 17.75	64.56 ± 15.98	81.36 ± 11.97	82.00 ± 13.02		
		PRP group	46.47 ± 16.50			33.47 ± 14.39	63.36 ± 11.73	77.65 ± 13.02	79.12 ± 13.42		
		*P* value	0.397			0.036	0.910	0.493	0.476		
	UCLA										
		Control group	16.78 ± 4.73			22.00 ± 4.01	25.94 ± 4.84	29.77 ± 4.36	30.83 ± 4.96		
		PRP group	15.89 ± 4.98			18.00 ± 7.47	26.27 ± 4.43	30.12 ± 6.04	31.78 ± 6.15		
		*P* value	0.558			0.061	0.827	0.708	0.579		
	ASES										
		Control group	49.60 ± 17.74			60.58 ± 16.33	71.75 ± 18.21	89.19 ± 10.73	89.92 ± 17.03		
		PRP group	43.95 ± 20.44			46.22 ± 20.06	72.63 ± 13.63	86.26 ± 19.95	87.61 ± 24.83		
		*P* value	0.343			0.031	0.530	0.712	0.744		
	DASH										
		Control group	45.69 ± 25.51			38.83 ± 20.14	23.80 ± 16.74	9.20 ± 9.87	8.48 ± 14.05		
		PRP group	52.85 ± 25.29			49.61 ± 23.12	24.85 ± 16.52	12.84 ± 18.89	13.19 ± 25.45		
		*P* value	0.369			0.166	0.703	0.588	0.473		
	SST										
		Control group	5.17 ± 2.99			5.40 ± 3.57	9.06 ± 5.45	10.64 ± 1.71	10.57 ± 1.73		
		PRP group	4.63 ± 3.29			4.40 ± 2.50	8.36 ± 2.25	9.59 ± 2.85	9.83 ± 3.31		
		*P* value	0.579			0.369	0.982	0.206	0.355		
	SPADI										
		Control group	46.25 ± 24.05			39.50 ± 23.02	25.71 ± 15.52	9.83 ± 10.59	10.08 ± 16.32		
		PRP group	54.47 ± 28.72			56.33 ± 23.97	28.69 ± 14.29	11.72 ± 18.22	12.03 ± 24.96		
		*P* value	0.318			0.045	0.745	0.869	0.673		

VAS: Visual Analog Score for Pain; PRP: platelet rich plasma; SER: strength in external rotation; UCLA: University of California; SST: Simple Shoulder Test; ASES: American Shoulder and Elbow Society; DASH: Disabilities of the Arm, Shoulder and Hand; SPADI: Shoulder Pain and Disability Index.
